# Genome-Wide Identification of the *COMT* Gene Family in *Juglans regia* L. and Response to Drought Stress

**DOI:** 10.3390/plants13192690

**Published:** 2024-09-25

**Authors:** Xiaolan Ma, Hongjia Luo, Jianhong Li, Zhiyue Wei, Yanlong Gao, Zhongxing Zhang, Yanxiu Wang

**Affiliations:** 1 College of Horticulture, Gansu Agricultural University, Lanzhou 730070, China; 15352454891@163.com (X.M.); 18894006572@163.com (Y.G.); 18394133890@163.com (Z.Z.); 2 Gansu Province Forestry Science and Technology Promotion Station, Lanzhou 730070, China; 18993049633@163.com (H.L.); 13919160940@163.com (J.L.); 3 Forestry and Grassland Bureau of Gansu Province, Lanzhou 730070, China; 17394361486@163.com

**Keywords:** walnut, *COMT*, lignin, drought stress

## Abstract

Caffeic acid O-methyltransferase (*COMT*), as a multifunctional enzyme involved in various physiological and biochemical processes in lignin metabolism, plays an important role in a plant’s response to stress. In this study, we isolated *COMT* family members from the walnut genome by bioinformatics and analyzed their physicochemical properties and their expression under drought stress to provide gene resources for drought resistance in walnut. The results showed that 33 *COMT* genes were identified from walnuts and distributed on different chromosomes. The molecular weight of proteins varies greatly. According to the phylogenetic tree, the family can be divided into seven subgroups, which are relatively conservative in evolution and closely related to *Arabidopsis thaliana*. Promoter analysis showed that the promoter of the walnut *COMT* gene contains rich cis-elements of plant hormone response and stress response, and the real-time fluorescence scale name can be significantly induced by drought stress. Compared with wild-type Arabidopsis, overexpression *JrCOMT19* significantly increased the enzyme activity (SOD, POD, and CAT) and proline content. Meanwhile, overexpression of *JrCOMT19* significantly increased the lignin content and expression of related genes. Therefore, *JrCOMT* plays an important role in responding to drought in walnuts, and overexpression *JrCOMT19* can improve the resistance to drought stress by increasing lignin content, antioxidant enzyme activity, and osmotic substance content.

## 1. Introduction

The growth and development of plants are often disturbed by the external environment, and how to improve the plant’s resistance to adversity is the focus of research [1]. Drought, as a common and serious natural stress, can inhibit plant growth and development, reduce crop yield, and cause plant death in severe cases [2]. As a characteristic industry of forest fruit widely cultivated in China, walnut is a tree with high economic and social value, and it is easily affected by drought stress, which will seriously affect the development of industry and economy [3]. Therefore, improving the drought resistance of walnuts is an urgent research task. Currently, the majority of research is concentrated on the development of resistant varieties through the mitigation of exogenous substances and molecular techniques; however, there remain numerous measures for further study and exploration.

As an important structural material in vascular plants, lignin not only enhances the strength of plant cells and tissues and facilitates water transport in plant tissues but also improves plant resistance. There is evidence that increasing the lignification degree and lignin content of plants is an effective measure against drought [4]. Caffeic acid O-methyltransferase (*COMT*) is an important methylase in the metabolic pathway of phenylpropane, which can catalyze the methylation of caffeic acid, 5-hydroxyphenylaldehyde to ferulic acid, mustard aldehyde [5], and also plays an important role in plant response to stress [6]. Studies have shown that overexpression of *SlCOMT1* (*Solanum lycopersicum* L.) can improve the drought resistance of tomatoes by increasing endogenous melatonin content, photosynthesis, and antioxidant capacity and also regulate abscisic acid (ABA) synthesis to promote stomatal closure and reduce water loss under drought stress [7]. Studies have found that silencing *GhCOMT28* (*Gossypium hirsutum* L.) can reduce melatonin levels in leaves, superoxide dismutase (SOD), peroxidase (POD), catalase (CAT) and increase the accumulation of H_2_O_2_, which reduced the drought tolerance of the plant [8]. In addition, most reports about the *COMT* gene are involved in the biosynthesis of S-lignin, and it has been reported in the studies of *Populus* L. [9], *Nicotiana tabacum* L. [10], *Medicago sativa* L. [11]. For example, the lignin content was significantly decreased after *COMT* gene inhibition in transgenic tobacco [12]. Therefore, the *COMT* gene is particularly important for plant resistance to biotic and abiotic stresses. However, the characteristics of the *JrCOMT* gene family and whether *COMT*-mediated lignin metabolism can respond to drought have not been reported. 

In this study, bioinformatics methods were used to identify the *COMT* gene family in walnuts, analyze its protein physicochemical properties, chromosome localization, conserved domain, and motif analysis, and study the expression pattern of the *COMT* gene under drought stress, aiming to explore the key *COMT* gene in walnut drought resistance response. It is helpful to further clarify the role of this gene family in the drought-resistant reaction process of walnuts and lay a foundation for the drought-resistant breeding of walnuts.

## 2. Results

### 2.1. Gene Identification of the JrCOMT Family

According to the specific Pfam of the *COMT* family, a total of 33 family members of *COMT* were isolated from the whole genome of walnuts, which were named *JrCOMT1*-*JrCOMT33* according to their unknown chromosomal characteristics. The ExPASy online tool was used to analyze the physicochemical properties of proteins, and the results showed that the amino acid size of *COMT* family members ranged from 185 to 1312 aa. The molecular weight ranges from 20,936.49 to 146,348.37 D. *JrCOMT12* has the lowest isoelectric point, while *JrCOMT7* has the highest. The hydrophilic index varies from −0.019 (*JrCOMT12*) to 0.309 (*JrCOMT7*). The aliphatic index ranges from 92.17 (*JrCOMT3*) to 107.45 (*JrCOMT7*) (Table 1).

### 2.2. Bioinformatics of JrCOMT Family

**Figure 1:**
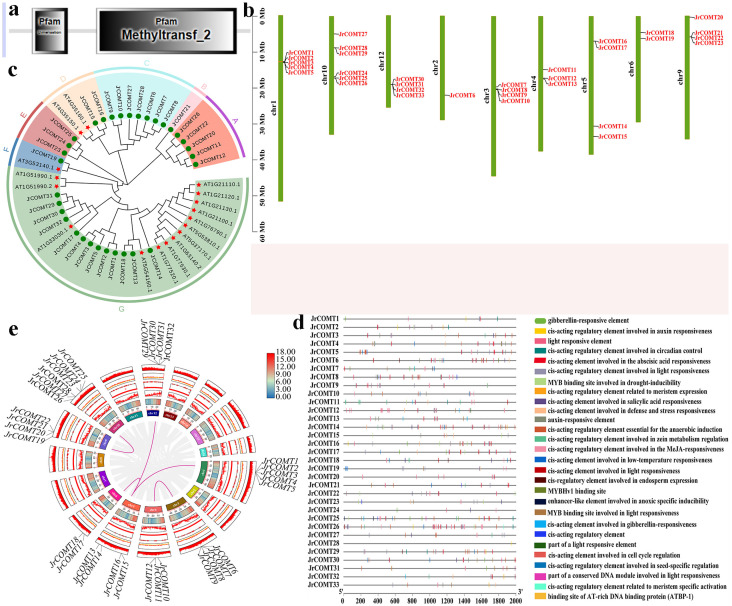
Bioinformatics analysis of *JrCOMT* gene family. (**a**) Domain specific to the COMT family. (**b**) Chromosome mapping of *JrCOMT* family genes. (**c**) Evolutionary tree analysis of *JrCOMT* and *AtCOMT* genes. The phylogenetic tree was generated using MEGA. The different colors in the figure represent the evolutionary tree groups (A–G) of *JrCOMT* and *AtCOMT*; the green dots represent the *JrCOMTs* gene, and the red five-pointed stars represent the *AtCOMTs* gene. (**d**) Cis-acting element analysis of *JrCOMT* family genes. Two kb 5′ upstream regions of all the identified *JrCOMT* genes were retrieved and analyzed through the PlantCARE database to identify the presence. The different cis-regulatory elements on each of the promoters were represented with different colors. (**e**) Intraspecific collinearity analysis of *JrCOMT* family genes. Sixteen chromosomes are represented in partial circles with different colors. Jrcomt genes in different chromosomes are indicated by black labels. Same colored lines connecting two chromosomal regions indicate the duplicated gene pairs in Medicago. The illustration was generated using CIRCOS-0.69-9 software.

Gene structure and conserved domain analysis showed that 33 JrCOMTs proteins have a C-terminal catalytic domain named Methyltransf-2 (PF00891), including SAM/SAH binding bags and substrate binding sites. SAM/SAH binding bags are highly conserved (Figure 1a). In order to understand the distribution of *JrCOMT* on chromosomes, 33 *JrCOMTs* were mapped, and the results showed that 33 *JrCOMT* family members were distributed on nine different chromosomes, of which six genes were distributed on Chr10 (Figure 1b).

In order to better understand the similarities and differences of COMT protein between walnuts and other plants, a phylogenetic tree was constructed using 33 walnut COMT protein sequences and 17 *Arabidopsis* protein sequences. Phylogenetic analysis of *JrCOMT* showed that 33 JrCOMT protein sequences can be divided into six groups, among which Group G contains 13 JrCOMT proteins, indicating that JrCOMT protein is relatively conserved in the evolutionary process and has a high degree of similarity (Figure 1c). 

Based on the 2000 bp sequences of 33 *JrCOMT* gene family members, the types and quantities of cis-acting elements in the promoter region were predicted to study the transcriptional regulatory factors of this gene family. As shown in the figure, the upstream 2000 bp sequence of promoters of *JrCOMT* gene family members are closely related to stress response (drought, light stress), hormone response (growth, salicylic acid, abscisic acid), defense and stress response, and so on, indicating that *JrCOMT* can participate in the regulation of a series of abiotic stress (Figure 1d). 

In order to study the evolutionary relationship of *JrCOMT* family genes in species, the collinearity between *JrCOMT* genes was plotted. The results showed that there were six collinearity relationships within *JrCOMT* family members (Figure 1e).

### 2.3. Expression Analysis of JrCOMTs under Drought Stress

**Figure 2:**
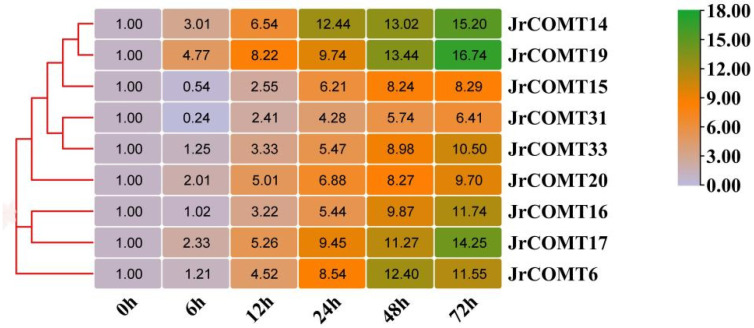
Heat map of *JrCOMT*s expression under drought stress. The expressive level of 9 different *JrCOMT*s genes at 0, 6, 12, 24, 48, and 72 h. Expression data were retrieved from genevestigator (https://genevestigator.com/gv/, accessed on 5 August 2024), and the heatmap was created with hierarchical clustering of Manhattan distance correlation using MeV 4.9.0 software package. A color scale is provided along with the heat map to recognize the differential pattern of expression.

According to the evolutionary tree analysis of *JrCOMT*s and *AtCOMTs*, *9JrCOMTs* closely related to *AtCOMTs* were selected for expression analysis under drought stress. The results showed that with the extension of drought stress time, the expression levels of 9 *JrCOMTs* genes gradually increased, in which the expression levels of *JrCOMT19*, *JrCOMT14,* and *JrCOMT17* were the highest, indicating that the *JrCOMT* gene plays an important role in responding to drought stress (Figure 2).

### 2.4. Bioinformatics Analysis of JrCOMT19 Gene

**Figure 3:**
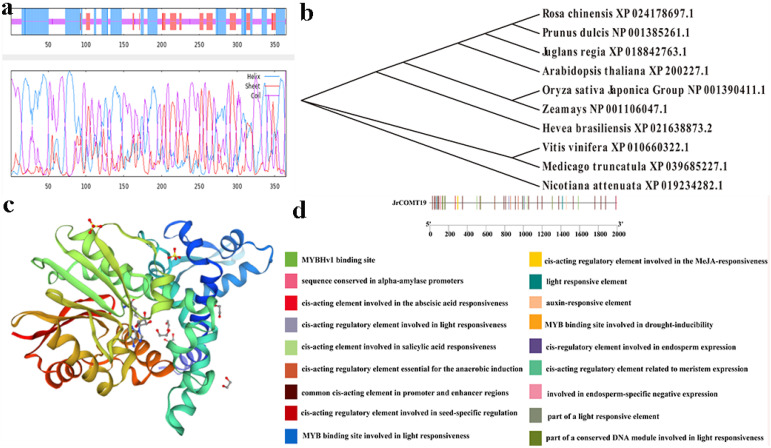
Bioinformatics analysis of *JrCOMT*19 gene. (**a**) Secondary structure of JrCOMT. (**b**) Evolutionary tree analysis of *JrCOMT* and other species. Protein sequences for other species were downloaded from NCBI. The phylogenetic tree was generated using MEGA. (**c**) The tertiary structure of JrCOMT. (**d**) Cis-acting element of *JrCOMT*.

As can be seen from Figure 3a, the secondary structure of JrCOMT19 mainly includes helix, sheet, and coil, and the predicted tertiary structure is consistent with that of the secondary structure (Figure 3c). Therefore, the predicted tertiary structure of the protein is considered reliable; it provides a preliminary basis for understanding the molecular function of the JrCOMT19 protein. 

The phylogenetic tree can be constructed to analyze the evolution of proteins more clearly. In this study, the MEGA7 software NJ method was adopted to construct the phylogenetic tree. The phylogenetic relationship between the JrCOMT19 protein and the other nine plants is shown in Figure 3b, which shows that the JrCOMT19 protein is a single branch and closely related to *Arabidopsis thaliana*, *Rosa chinensis,* and *Prunus dulis*, suggesting that these proteins are functionally similar.

To further determine the function of the *JrCOMT19* gene in stress response, the sequencing results were submitted to the PlantCARE website for the prediction of cis-acting elements. The results show that the promoter region of *JrCOMT19* contains auxin, abscisic acid, salicylic acid computing response elements, and drought, light stress response elements, indicating that *JrCOMT19* plays an important role in responding to stress (Figure 3c).

### 2.5. Cloning of JrCOMT19 Gene and Identification of Transgenic Arabidopsis thaliana

**Figure 4:**
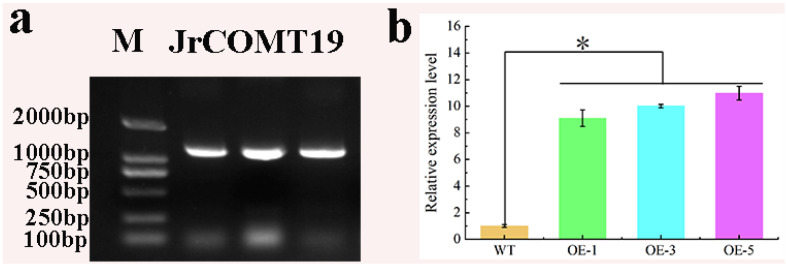
Amplification and expression of *JrCOMT*19 gene in transgenic *Arabidopsis thaliana*. (**a**) Glue map of *JrCOMT*19 gene clone. (**b**) Expression levels of *JrCOMT* gene in WT and transgenic *Arabidopsis thaliana*. “*” means that the difference between samples is significant at the level of 0.05 (*p* < 0.05). Data are means of three replicates with SE. Values not followed by the same letter indicate significant differences between treatments, according to Duncan method of single-factor ANOVA (*p* < 0.05).

The target fragment of about 1098 bp was amplified using the cDNA of Liaohe-4 seedling as the template (Figure 4a). The purified and recovered target strip was connected to the pMD19-T vector, and the positive clone was detected by primers and sequenced. Sequencing analysis showed that the fragment was identical to the target fragment in the Walnut genome database.

*Arabidopsis thaliana* was infected by agrobacterium transformation, and the T0 generation *Arabidopsis* seeds obtained from the first infection were screened for three successive generations until homozygous transgenic *Arabidopsis thaliana* was obtained. Transgenic *Arabidopsis thaliana* was extracted for real-time fluorescence quantitative analysis, and the results showed that the expression level of the *JrCOMT*19 gene in transgenic *Arabidopsis thaliana* was significantly higher than that of wild-type (Figure 4b).

### 2.6. Phenotype Observation and Index Determination of Arabidopsis thaliana with 10% PEG 6000

**Figure 5:**
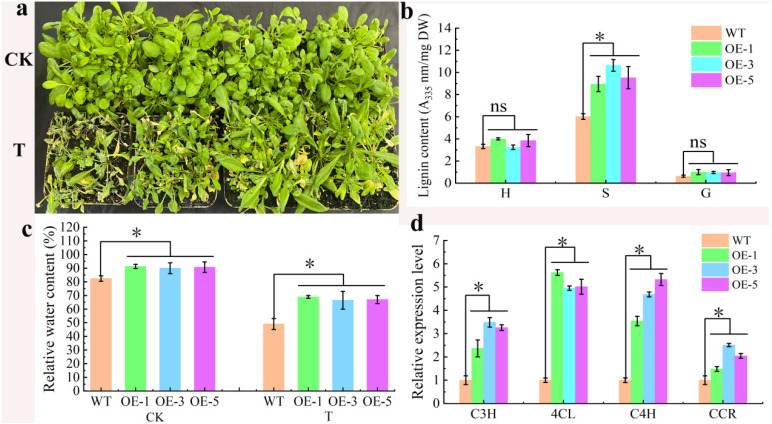
The phenotypes of *JrCOMT*19-OE and wild-type (WT) *A. thaliana* under normal conditions (CK) and drought stress (T). (**a**) Phenotypes of wild and transgenic *Arabidopsis thaliana* under drought stress. H, S, and G represent three different types of lignin. (**b**) Lignin content. (**c**) Relative water content. (**d**) Relative expression level of genes resulting in lignin. “ns” means that the difference between samples is not significant, and “*” means that the difference between samples is significant at the level of 0.05 (*p* < 0.05). Data are means of three replicates with SE. Values not followed by the same letter indicate significant differences between treatments, according to Duncan method of single-factor ANOVA (*p* < 0.05).

In order to determine the response of *JrCOMT*19 to drought stress, three strains of *Arabidopsis JrCOMT19*-OE (OE-1, OE-3, OE-5) and WT were cultured under normal stress and drought stress for 20 days, respectively. As shown in Figure 5a, there was no difference between WT and transgenic *Arabidopsis thaliana* under normal growth conditions, both of which showed a good growth state, while under drought stress, transgenic *Arabidopsis thaliana* showed a better growth state, and WT leaf yellow flowers were serious. We measured relative water content and found that transgenic *Arabidopsis* was better able to retain water under drought stress (Figure 5b).

The results of lignin content determination showed that overexpression of *JrCOMT*19 significantly increased the S-lignin content under drought stress, while there was no significant difference between H-lignin and G-lignin content (Figure 5c). In addition, real-time fluorescence quantitative results showed that overexpression of *JrCOMT*19 could significantly induce the expression of lignin-related genes (Figure 5d).

**Figure 6:**
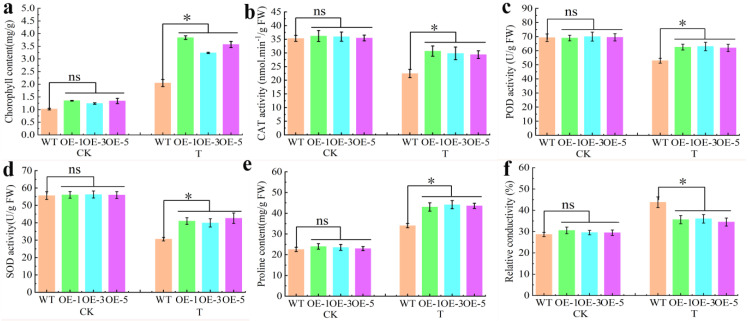
Physiological indices of *JrCOMT*19 and wild-type (WT) *A. thaliana* under normal conditions (CK) and drought stress (T). (**a**) The Chl content. (**b**) The CAT activities. (**c**) The Pro content. (**d**) The POD activities. (**e**) The SOD activities. (**f**) The REC conductivity. “ns” means that the difference between samples is not significant, and “*” means that the difference between samples is significant at the level of 0.05 (*p* < 0.05). Data are means of three replicates with SE. Values not followed by the same letter indicate significant differences between treatments, according to Duncan method of single-factor ANOVA (*p* < 0.05).

In order to better understand the response of the *JrCOMT*19 gene to drought stress, we determined a series of drought resistance indicators of *Arabidopsis thaliana* under normal conditions and drought stress, and the results showed that overexpression of *JrCOMT19* significantly increased chlorophyll content under drought stress compared with the WT (Figure 6a). The results of the antioxidant oxidase test showed that there was no significant difference in CAT, POD, and SOD activities between WT and transgenic *Arabidopsis thaliana* under normal growth conditions, while CAT, POD, and SOD activities in overexpressing *JrCOMT19* were significantly higher than those of WT under drought stress (Figure 6b–d). The results of proline content determination showed similar results (Figure 6e).

The relative conductivity of WT was significantly higher than that of transgenic strains under drought stress, indicating that overexpression of *JrCOMT19* could reduce the damage to *Arabidopsis thaliana* under drought stress (Figure 6f). These results suggested that overexpression of *JrCOMT*19 enhances *Arabidopsis* tolerance to drought stress.

## 3. Materials and Methods

### 3.1. Genome Identification of JrCOMT Genes in Walnut

The protein sequence of COMT family genes was obtained from Walnut genome database (http://xhhuanglab.cn/data/juglans.html, accessed on 5 August 2024), and the *Arabidopsis* COMT protein data were obtained from TAIR (https://www.arabidopsis.org, accessed on 5 August 2024) [13]. The latent Markov model (PF00891) was used for preliminary screening of candidate genes [14]. The ExPASy was used to analyze the physical and chemical properties of members of the JrCOMT family [15].

### 3.2. Analysis of the JrCOMT Family Members

Software was used to predict the conserved motifs of *JrCOMT* family and map the gene structure and conserved motifs of *JrCOMT* family members. MapChart 2.2 software and TBtools were used for chromosome mapping [16]. DNAMAN 8.0 software was used to carry out multiple sequence alignment. The phylogenetic tree was constructed with MEGA-X technique, and the possible biological functions of these family members were predicted [17]. The gene information of walnut family was analyzed by McscanX 2018 software of Linux system, the replication relationship between genes was obtained by TBtools-1-09876, and the collinearity of walnut was analyzed by Circos and McscanX 2018 software, respectively [18].

### 3.3. Expression Characteristics of JrCOMT Gene Family under Drought Stress

The 4-week-old Liaohe No. 4 tissue culture seedling in the tissue culture room of the Department of Fruit Science, College of Horticulture, Gansu Agricultural University, was used as the material. In vitro seedlings of walnut with robust growth and consistent growth were selected to simulate drought stress with 10% PEG 6000, and normal growth seedlings were used as controls, with 15 plants per treatment and 3 biological replicates. After 0, 6, 12, 24, and 48 h of stress treatment, leaves were rapidly frozen in liquid nitrogen and stored at −80 °C for gene expression analysis.

Use the kit to extract RNA from the remaining branches for subsequent quantification. The level of *JrCOMT* family expression was determined using quantitative real-time (qRT) PCR. Simply, the Ct value of *JrCOMTs* is compared to that of the internal reference gene (18s) to derive the ΔCt value. Subsequently, the ΔCt value with drought stress is contrasted with that under normal conditions to obtain the ΔΔCt value. Finally, relative expression levels are calculated using the formula 2^−ΔΔCt^. The 18s gene was chosen as reference gene [19]. Additionally, the primers for their entirety are displayed in Table 2. The average of three separate experiments served as the foundation for the outcomes.

### 3.4. Cloning and Bioinformatics Analysis of JrCOMT19

We took *JrCOMT19*, which has the highest expression level under drought stress, as the target gene and Liaohe-4 seedling as the test material to extract RNA for future use. In addition, the bioinformatics analysis of *JrCOMT19* gene was carried out. All specific methods refer to Ma [19].

### 3.5. Agrobacterium Mediated Transformation of Arabidopsis thaliana and Determination of Related Physiological Indexes

The genetic transformation of *Arabidopsis* was carried out according to the method of Ducloy [20]. It was treated with 75% ethanol for 5 min, 2.6% sodium hypochlorite (NaOCl) for 10 min, and washed with deionized water 3 times, finally grown in MS medium (MS + 30 g/L sucrose + 8 g/L Agar, pH = 5.8–6.0). After green seedlings were grown, they were moved to the substrate (peat: perlite: vermiculite = 3:1:1) for growth and infected *Arabidopsis* by infecting inflorescence at flowering time, infecting once a week, a total of 3–4 times. The infected *Arabidopsis* seeds were planted on MS medium containing 30 mg/L Kan for 3 successive generations to ensure the homozygous transgenic *Arabidopsis*.

The lignin content was determined by extraction of mercaptoacetate lignin [21], the relative conductivity was determined by conductivity meter [22], the proline (Pro) content was determined by ninhydrin method [23], and the relative water content of leaves was determined by weighing method [24]. The activities of superoxide dismutase (SOD), peroxidase (POD), and catalase (CAT) were measured using a spectrophotometer and a kit from Suzhou Kaiming Biological Co., Ltd. (Suzhou, China).

### 3.6. Statistical Analysis

The means and standard errors (SE) of three distinct replicates were used to display the experimental data. SPSS version 22.0 (IBM, Armonk, NY, USA) was used for statistical analyses; the map was drawn with Origin 9.1.

## 4. Discussion

At present, the most effective way to alleviate drought stress is to use molecular means to mine plant resistance genes, which has important research value and broad application prospects for improving plant abiotic resistance [25]. Some studies have found that overexpression of *MdTPR16* can reduce the oxidative damage of apple cells by reducing electrolyte leakage and malondialdehyde content, thereby improving the resistance to drought stress [26]. Studies have shown that *ZmMYB56* mutation leads to increased stomatal conductance and rapid water loss in isolated leaves, resulting in severe drought sensitivity [27]. Although more genes have been reported, there are still many genes to be discovered.

Lignin is considered to be the key for plants to evolve from aquatic plants to adapt to terrestrial ecosystems [28], and lignin pathways have been extensively involved in plant responses to biological stresses [29]. *COMT* is a key enzyme in the lignin metabolic pathway and plays an important role in abiotic stress [30]. However, the response of *COMT* to stress has not been reported in walnuts. 

In this experiment, the bioinformatics analysis and genetic transformation of *JrCOMT* from Liaohe were conducted to verify its response to drought stress. A total of 33 *COMT* genes were identified based on the whole genome information of walnuts, and their physicochemical properties, conserved domains, and evolutionary analysis indicated that the function of this family was conservative in the process of plant evolution and individual differences may be caused by evolution [31]. In addition, proteins with similar conserved motifs and conserved domains in *JrCOMT* members are grouped together, so it is inferred that genes in the same phylogenetic group have similar structural, functional, and evolutionary characteristics [32]. In plants, gene duplication events play a key role in the evolution of gene families [33]. The duplicated *JrCOMT* gene pairs were mainly distributed on 7 chromosomes (Chr 1, Chr 3, Chr 4, Chr 5, Chr 6, Chr 9, and Chr 10), which indicated that segmental duplication events occurred on 7 of 16 chromosomes in *Juglans regia* L. Therefore, segmental duplication phenomena played a more important role in *JrCOMT* gene duplication [34]. To further investigate the *JrCOMT* function, the cis-acting elements binding transcription factors to regulate genes were analyzed. Cis-acting elements in *JrCOMT* promoters mainly consist of plant hormone (gibberellin, auxin, salicylic acid) and adversity (drought, low temperature) response element, indicating that they were important in hormone and stress-mediated events and likely to affect the function of *JrCOMT* genes.

On the other hand, we obtained *JrCOMT* transgenic *Arabidopsis thaliana* and observed and determined the phenotype and related indicators under drought stress. The transgenic *Arabidopsis* maintained a good growth state under drought stress, which may be due to the overexpression of *JrCOMT19*, which prevented water loss and promoted enzyme activity and expression of related resistance genes in *Arabidopsis* [35]. When plants are subjected to biological stress, they mainly change the expression of genes related to the lignin pathway, promote the increase in enzyme activity, and accumulate different metabolites to fight against biological stress. By measuring the lignin content, it was found that the transgenic *Arabidopsis thaliana* with high resistance to drought had higher lignin content, quantitative analysis of related genes in the lignin synthesis pathway of *Arabidopsis thaliana* with the *JrCOMT19* gene showed that *C3H*, *4CL*, *CCR,* and *C4H* genes were significantly upregulated. Therefore, we speculate that overexpression of *JrCOMT19* increases lignin content by inducing lignin gene expression to improve resistance to drought [36].

In addition, *COMT* is involved in the synthesis of melatonin [37], an indoleamine small molecule compound that is widely found in plants and animals and is a biological regulator of circadian rhythms and plant growth, development, aging, and stress responses [38]. Moreover, it was found that the application of melatonin increased the expression of *COMT*, increased the lignin content by regulating the metabolism of phenylpropanoid, and enhanced the resistance of cotton and tomato to Botrytis grisea [39], indicating that lignin and melatonin biosynthesis were related. Therefore, we speculated that overexpression of *JrCOMT19* may also promote the synthesis of melatonin to improve the activity of antioxidant enzymes, regulate the release of reactive oxygen species (ROS) in cells, enhance the antioxidant defense ability, reduce chlorophyll loss and oxidative damage, and regulate the transcription level of resistance-related genes, thus improving the drought resistance of *Arabidopsis*. Our subsequent determination of drought resistance indicators confirmed this conjecture. Although melatonin and lignin play a role in plant growth and development and stress response, the specific molecular mechanism of their interaction remains to be further explored.

When plants are subjected to low-temperature stress, a large number of ROS are produced in the cells, the cell membrane is damaged, the intracellular soluble substances are extravasated, the relative electrical conductivity of seedlings roots is increased, and a large amount of MDA accumulation will lead to the loss of plant cell vitality [40]. At the same time, stress induces the activity of an antioxidant enzyme system in plants, which can resist the accumulation of reactive oxygen species in cells, relieve membrane lipid peroxidation, and resist damage to plants caused by stress [41]. In this study, overexpression of *JrCOMT* decreased the relative conductivity of *Arabidopsis thaliana* under drought stress, and the activities of SOD, POD, and CAT increased, indicating that *JrCOMT* could alleviate the damage of drought stress on the *Arabidopsis* membrane. 

In addition, proline, as one of the important osmoregulatory substances, maintains the normal growth and metabolism of plants by maintaining the water content and turgor potential of cells [42]. In this study, it was found that overexpression of *JrCOMT* significantly increased the proline content and relative water content under drought stress, indicating that regulating the synthesis and accumulation of osmotic substances may also be one of the strategies of *JrCOMT* regulating *Arabidopsis* drought alleviation. However, the downstream pathways regulated by the *JrCOMT19* gene under abiotic stress still need further exploration. Therefore, more in-depth studies should be conducted in the future to investigate the molecular mechanisms underlying salt and drought tolerance regulated by the *JrCOMT19* gene, such as the regulatory relationships between proteins or between proteins and nucleic acids, so as to systematically analyze the molecular pathways through which the *JrCOMT19* gene regulates plant phenotypes.

## 5. Conclusions

In summary, we confirm that *JrCOMT* can improve the resistance of transgenic *Arabidopsis thaliana* to drought stress and reveal its mechanism in terms of osmotic regulation and antioxidant system under drought stress. Specifically, overexpression of *JrCOMT* increased the activity of SOD, POD, and CAT enzymes under drought stress, effectively prevented water loss, decreased relative conductivity, and increased lignin content and expression of related genes to increase drought resistance.

## Figures and Tables

**Table 1:** Analysis of physicochemical properties of *JrCOMT* family.

Accession No.	Gene Name	Size (aa)	Molecular Weight (D)	Isoelectric Point	Grand Average of Hydropathicity (GRAVY)	Aliphatic Index
JreChr01G10207	*JrCOMT1*	366	40,160.30	6.06	−0.104	83.39
JreChr01G10220	*JrCOMT2*	378	41,851.29	6.07	−0.117	83.84
JreChr01G10229	*JrCOMT3*	368	40,843.32	5.75	0.046	92.17
JreChr01G10230	*JrCOMT4*	368	40,824.26	5.95	−0.013	89.91
JreChr01G10232	*JrCOMT5*	343	37,863.87	6.32	−0.028	88.98
JreChr02G10946	*JrCOMT6*	340	38,366.60	9.00	−0.127	87.44
JreChr03G10911	*JrCOMT7*	306	33,721.43	9.22	0.309	107.45
JreChr03G10972	*JrCOMT8*	327	35,517.03	5.99	0.097	97.52
JreChr03G10984	*JrCOMT9*	355	38,815.00	6.16	0.097	96.99
JreChr03G10985	*JrCOMT10*	355	38,797.97	6.07	0.109	97.01
JreChr04G10415	*JrCOMT11*	355	39,716.25	5.77	0.009	95.61
JreChr04G10555	*JrCOMT12*	379	42,720.38	5.26	−0.019	96.25
JreChr04G10568	*JrCOMT13*	367	41,338.97	5.53	0.029	98.34
JreChr05G11501	*JrCOMT14*	365	39,962.32	5.88	0.029	90.55
JreChr05G11714	*JrCOMT15*	372	41,426.89	5.84	−0.046	96.96
JreChr05G12697	*JrCOMT16*	354	39,010.38	5.30	−0.026	92.06
JreChr05G12699	*JrCOMT17*	1312	146,348.37	6.00	−0.036	95.71
JreChr06G11870	*JrCOMT18*	373	40,786.08	5.95	0.028	92.60
JreChr06G12011	*JrCOMT19*	365	39,746.97	5.42	0.064	91.89
JreChr09G11939	*JrCOMT20*	359	39,799.46	5.59	−0.207	85.01
JreChr09G12161	*JrCOMT21*	363	40,507.72	5.67	−0.076	94.79
JreChr09G12162	*JrCOMT22*	292	33,269.04	9.02	0.049	87.50
JreChr09G12163	*JrCOMT23*	361	40,904.29	5.91	−0.162	88.01
JreChr10G10468	*JrCOMT24*	185	20,978.67	6.06	−0.007	96.38
JreChr10G10485	*JrCOMT25*	365	40,194.37	5.31	−0.016	98.08
JreChr10G10488	*JrCOMT26*	365	40,209.45	5.32	0.010	99.40
JreChr10G11800	*JrCOMT27*	486	54,381.39	5.82	−0.227	94.22
JreChr10G12207	*JrCOMT28*	192	20,936.49	6.18	0.105	100.05
JreChr10G12210	*JrCOMT29*	355	38,962.39	6.04	0.103	96.70
JreChr12G10775	*JrCOMT30*	369	40,530.62	5.59	0.018	95.58
JreChr12G10776	*JrCOMT31*	369	40,443.67	5.62	0.054	95.37
JreChr12G10777	*JrCOMT32*	369	40,364.29	5.69	−0.017	92.44
JreChr12G10778	*JrCOMT33*	207	23,474.06	8.75	−0.197	82.37

**Table 2:** List of primers for real-time fluorescence quantification and gene cloning.

Purpose	Primer	Primer Sequence (5′–3′)
Reference gene	*18S*	F:ACACGGGGAGGTAGTGACAA R:CCTCCAATGGATCCTCGTTA
Quantitative real-time PCR	*JrCOMT6*	F:AGGAGGAAGAGTACATTGAATGGTTTG R:AGCCATGCCGACGGACAC
	*JrCOMT14*	F:CAACAGAGCCTACGGAATGACAG R:TGGTTTGACATTGCTTGGTTGAATAC
	*JrCOMT15*	F:TGAATGTATTCTTCCAGTAGCACCAG R:AACTCCTTCTCTGTCCTCTCCTTC
	*JrCOMT16*	F:CCGTCTCCTCAATGAAGCAATGG R:CCTCTGGACAACCTTGAAGAATCG
	*JrCOMT17*	F:ATACAACAAGCCATCCGCATCTC R:CATCTTCTTATCTACACTGCTCCTCTC
	*JrCOMT19*	F:TCTCTGACGAAGAAGCCAACCTC R:GCCCAGCCTTTGCGATGATG
	*JrCOMT20*	F:GGGAAGGGATCAACTTTGACTTACC R:TGGCATCAGCAGAAGGAATAGAATG
	*JrCOMT31*	F:GATCTTGGTGTGCTTGTGATTATTGG R:GGTTGGCGTTGCTGTGAGTG
	*JrCOMT33*	F:GTTGTCAAGTGTCGGCAGTTCC R:CACATCCACAAGCGACTTCAGG
Gene clone	*JrCOMT19*	F:ATGGGCTCCACCGGAGAA R:TCAAAGCTTTTTAATGAATTCCATG
Quantitative real-time PCR	*C3H*	F:GAACTGATTGGAAAGCTCGGAAACATC R:GCGAGTTCAACGGAGTGCTGTAG
	*C4H*	F:ACACCATCATCGTCATCACACTCATC R:TCCAAGCTCTTCTTCACCAGTTGC
	*4CL*	F:ACACCATCATCGTCATCACACTCATC R:TCCAAGCTCTTCTTCACCAGTTGC
	*CCR*	F:CGAGCCACCCAAGCAAGACTATATC R:ACTTTCATCCTTTCGCTGATCTTCTCTC

## Data Availability

Data are contained within the article.
